# 

**Published:** 1888-03

**Authors:** 


### INDEX.

A

A systematic stretching for shortening of the broad and utero sacral ligaments 75.
Abdominal tumors in the Negro race 235.
Abcess of the cerebellum following suppurative otitis media 231. Perineal—complicating stricture of the urethra 14. Tuberculous—implicating the elbow joint 219,of the tarsus 221.
Adulteration of drugs 376.
Agaricine in the sweating of phthisis 31.
Alabama—new codeism in 98-119—medical laws of 305.
Amputation through doubtful tissues 759.
An anomalous case discovered in the female sex 385.
Anatomical Board of Georgia 253.
Aneurism of the ascending aorta—report of a case—involving the innominate secondarily, operated upon by simultaneous ligation of the common carotid and subclavian—cure 677.
Antipyrine 145.
Antipyretics—fever and the use of 513.
Antisepsis in gynecology 154-212.
Anus—fissure of 327.
Ascites—cirrhosis of the liver with marked 49.
Asthmatic bronchitis 617.
Asylums—inebriate 638.
Atlanta—city hospital for 37, Society of Medicine 316.
Atlanta Medical College 120, annual address delivered to the graduating class 65
Auditory canal—furuncular inflammation of the external 82.

B

Babies—the 312.
Bacilli—tubercle—staining of 200.
Book Reviews.—A manual of the physical diagnosis of thoracic diseases 116, A manual of diseases of the nervous system 580, Annual of the Universal Medical Sciences 629, A practical treatise on impotence, sterility and allied diseases of the male sexual organs 117, A practical treatise on diseases of the skin 310, A treatise on human physiology 503, Cyclopædia of Obstetrics and Gyneæcology 372, Differential Diagnosis 116 Disinfection and Disinfectants 635, Elementary Microscopical Technology 117, Hand Book of Geographical and Historical Phthisisiology 633, Ptomaines and Leucomaines 631,Photographic illustrations of skin diseases 311 Inebriety 630, Reference Hand Book of the Medical Sciences 441, Rules of Aseptic and Antiseptic Surgery 112, Sexual Impotence in the Male and Female 117, Surgical Diseases of the Genito-Urinary Organs, including Syphilis 310, Syphilis 114, (1) On a new treatment of Chronic Metritis,and especially of Endo-Metritis with intra-uterine chemical galvano cauterizations 373, Transactions of the Medical Association, State of Alabama 631, Transactions of the Medical Society, State of Pennsylvania 632, The ear and its diseases 634, The applied anatomy of the nervous system 636. The pathology, diagnosis and treatment of the diseases of women 374, The Physician's Pocket Day Book 637.
Brain disease 233.
Breast—cancer of the 230.
Bronchitis—asthmatic 617.

C

Cæsarean section 237.
Calcul'—meat diet as a cause of 424.
Calculus—symptoms of—attending contracted meatus 16.
Cancer—of the breast 230, of the prostate 49.
Carcinoma uteri—successful vaginal hysterectomy for 681.
Caries—unusual result of long standing tarsal 765.
Cascara Sagrada—severe effects of 24.
Catarrh—constipation a cause of 740.
Cellulitis—chronic pelvic, and the conditions which simulate it 715.
Cerebellum—abcess of, following suppurative otitis media 231.
Cervical canal—fibrous tumor of 300.
Cervix uteri—extensive laceration of 107.
Chancroid and gonorrhœa—the evolution of 431.
Chloroform anæsthesia and narcosis as a remedy in cerebro spinal meningitis 602.
Chorea of the soft palate caused by the hypertrophy and hyperesthesia of the mucuous membrane covering both inferior turbinated bones 455.
Circumcision—Two cases of reflex irritation cured by 470.
Cirrhosis of the liver with marked ascites 49.
Club-foot—treatment of, by means of the Thomas wrench, with report of cases 749.
Cocaine 25, some unpleasant effects of 270.
Coccygeal region—dermoid cysts of 651.
Convulsions—puerperal 756.
Correspondence.—A new procedure in retention of urine 104, A specimen of exostosis of the nasal septum 438, Louisiana State Medical Society *vs.* the editorial staff of the New Orleans *Medical and Surgical Journal* 364, New Codeism in Alabama 98, New York letter 51, 96, 170, 243, 302, 361, 435, 496, 570, 626, 703, 769, Multiple neuritis 497, The congress of American physicians and surgeons 573, Quarantine conference 771, Religious newspapers and quackery 308, Tennessee State Medical Society 182, The Medical Association of Alabama 175, The Medical Association of Georgia 179, The medical laws of Alabama 305, The tariff on Surgical instruments 54.
Copperas—supposed case of poisoning by 198.
Corneal ulcer—treatment of, with eserine 355.
Counter prescribing 377.
Cranial surgery 672.
Cranium—two cases of compound fracture of the, with depression 449.

Cut throat—a case of 670.
Cystic tumor—removal of, of labium majus 12.

D

Deltroid muscle—complete paralysis of 109.
Dermatology—electrolysis in 287.
Dermoid cysts of the coccygeal region 651.
Disease—is the germ theory of, rational 129.
Disinfectant—peroxide hydrogen as a germicide and 9.
Dislocation and fracture of humerus 321.
Drugs—Adulteration of 376.
Dysentery (so called) entero colitis 282.

E

Electrolysis in dermatology and in some other departments of practical surgery 287.
Electrolysis in morbid alterations that are produced in the prostate by gonorrhœa of the urethra 686.
Empyema—a case of 527, the treatment of 389.
Entero colitis 282.
Epilepsy—a case of, cured (apparently) by the correction of an error of refraction 206. Traumatic, operative procedures in 663.
Erysipelas—pilocarpus in 396.
Eureka 252.
Exostosis of the nasal septum, a specimen of 438.

F

Female sex—an anomalous case discovered in the 385.
Fever and the use of antipyretics 513.
Fever—typhoid 49. In childhood 485. Practical experience in, as it occurs in Alabama 273-331.
Fissure of the anus 327.
Fistula—essence of turpentine as an injection in 30.
Follicular pharyngitis 622.
Foreign bodies in the nose 742.
Fracture of the cranium with depression; two cases of compound 449, and dislocation of the humerus 321, of the skull 321.
Fracture of the forearm 658.
Fracture of patella treated by wiring 452.
"Frederick the Noble," the case of 581.
Furnuncular inflammation of the external auditory canal 82.

G

Georgia Medieal Society and the tariff 57.
Germ theory—is the, of disease rational 129.
Germicide—peroxide of hydrogen as a disinfectant and 9.
Glasses—the necessity for wearing, and a plea for the early recognition of the conditions requiring their use 520.
Gynecology—antisepsis in 154-212.

H

Hæmathorax—a case of, following an incised wound of the thoracic wall 465.
Hæmatocele—a case of pelvic 32.
Hæmaturia—a clinical lecture on 87.
Hemorrhoids—how shall we treat? 1; the treatment of 257.
Hernia—strangulated, herniotomy 203.
Humerus—fracture and dislocation of 321.
Hydrogen peroxide as a disinfectant and germicide 9.
Hymen—the 485.
Hypnotic—a new, sulfonal 375.
Hysterectomy for malignant diseases of the uterus 532. Vaginal, report of three cases 398.

I

Inebriate asylums 638.
Intestinal obstruction, chronic, report of case and operation 597.
Intubation and tracheotomy 137.
Items 59, 86, 94, 118, 124, 173, 184, 255, 319, 378, 410, 447, 483, 494, 508, 537, 569, 616, 625, 640, 708, 775.

K

Kidneys and bladder—tubercular diseases of 350.
King's Daughters' Hospital 507.
Knife—the, in uterine surgery, its use and abuse 266.

L

Labor—a case presenting unusual complications 110.
Labium majus—removal of cystic tumor of 12.
Laparotomy for diseases of the liver 226, purulent peritonitis 358.
Lithotomy—nephro 47.

M

Meatus—contracted, attended by marked symptoms of calculus 16.
Meat diet as a cause of calculi 424.
Medical Association of Georgia 119.
Medical colleges of the city, their closing exercises 120.
Medico-legal interest—two cases of 728.
Meningitis—cerebro spinal, chloroform anæsthesia and narcosis as a remedy in 602.
Menstruation—vicarious 109.
Mercury—salicylate of, a practical investigation of the therapeutic value of 194.
Multiple neuritis 497.
Muscle—deltoid, complete paralysis of 109.
Myxomata of the orbit with colloid degeneration 530.

N

Nasal septum—a specimen of exostosis of the 438.
Nephro-lithotomy 47.
Neuralgia—sciatic, the external application of sulphur in 340.
Neuritis, multiple 497.
Newspapers—religious and quackery 308.
Nose—foreign bodies in the 742.
Notice 318.

O

Obstruction—chronic intestinal, report of case and operation 597.
Orbit—Myxomata of the, with colloid degeneration 530.
Oesophageal stricture 467.
Opthalmia—sympathetic 767.

P

Papilloma—mucous, two cases of, with carcinomatous degeneration of the rectum 17.
Papillomata—sub-glottic 592.

Para-metritis—the treatment of, by dilating and curetting the uterus 472.
Patella—fractured, treated by wiring 452.
Pelvic cellulitis—chronic, and the conditions which simulate it 715.
Pelvic hæmatocele, a case of 32.
Peritonitis—purulent, laparatomy for 358.
Personal:—Drs. B. W Bizzell 379, J. H. Branham 185, D. J. Cain 59, J. D. S. Davis 702, W. E. B. Davis 702, J. A. Fitzhugh 186, J. T Galt 184, A. W. Griggs 59, M. B. Hutchins 124-256, J. P. Logan 382, F. W. McRae 124-448, H. V. M. Miller 382, K. P. Moore 185, T. O. Powell 59, S. W. Pryor 124, G. H. Rohe 185, F. O. Stockton 255, H. B. Tate 59, A. VanderVeer 185, W. H. Way 775, A. G. Whitehead 59, Mr. E. B. Treat 59.
Pharyngitis—follicular 622.
Phthisis—agaricine in the sweating of 31.
Physician—the embryo—as a specialist 343,
Pilocarpus in erysipelas 396.
Pneumonia—a clinical lecture on 16.
Poisoning by copperas—a supposed case of 198.
Pregnancy—Hegar's test for 357; uncontrollable vomiting in; cured by alimentation with a tube in the œsophagus 30
Prescribing—counter 377.
Prostate—cancer of 49.
Psoriasis—a clinical lecture on 35.
Puerperal convulsions 757.
Pulmonary tuberculosis—on the selection of a climate for patients with 475.

Q

Quackery—religious newspapers and 308.
Query 639.

R

Rectum—two cases of mucous papilloma with carcinomatous degenerations in the 17.
Refraction—a case of epilepsy cured (apparently) by the correction of an error of 206.
Religious newspapers and quackery 308.
Retention of urine—a new procedure in 104.
Retrospect 775.

S

Salycilate of mercury—a practical investigation of the therapeutic value of 193.
Sciatic neuralgia—the external application of sulphur in 340.
Selections 246, 343, 411, 472, 475, 539, 560.
Severe effects of cascara sagrada 24.
Shortening of the broad and utero-sacral ligaments—a systematic stretching for 75.
Shock 560.
Skull—fracture of 322.
Society reports:—Allegheny County Medical Society 490-567-758, Atlanta Society of Medicine 105-755, Chicago Medical Society 43, Chicago Pathological Society 350-422, Clinical Society of Maryland 224-355.
Southern Medical College 122.
Southern Surgical and Gynecological Association 446-706; President's address 698.
Specialist—the embryo physician as a 342.
Strangulated hernia--herniaotomy 203.
Stricture—œsophageal 467, of the urethra complicated by a perineal abcess 14.
Sub-glottic papillomata 592.
Sulfonal—a new hypnotic 375.
Superinvolution of the uterus following trachelorrhaphy 538.
Surgical instruments—the tariff on 54.
Syphilis--successful treatment of 167.

T

Tait's operation 756.
Tariff on surgical instruments 54.
Tetanus—the present state of 43.
Throat cut—a case of 670.
The case of "Frederick the Noble" 581.
The Journal—new series, volume 5, 56.
Trachelorrhaphy—superinvolution of the uterus following 538.
Tracheotomy and intubation 137.
Traumatic epilepsy--operative procedure in 663
Tubercle bacilli—staining of 200.
Tubercular disease of the kidneys and bladder 350.
Tuberculous abcess—implicating the elbow joint 219, of the tarsus 221.
Tumor—cystic, removal of—of labium majus 12; fibrous, of the cervical canal 300.
Tumors—abdominal, in the Negro race 235.
Turpentine--essence of, as an injection in fistula 30.
Two cases of medico-legal interest 728.
Typhilitis 354.
Typhoid fever 49, in childhood 485, practical experience in, as it occurs in Alabama 273-331.

U

Ulcer—corneal, treatment of, with eserine
Urethra--stricture of, complicated by perineal abscess 14.
Urethra and testes—what shall the surgeon do with the, in cases of amputation and ablation of the penis 730.
Urethrotomy—internal, with report of cases 587.
Urine—retention of, a new procedure in 104.
Uterus—hysterectomy for malignant disease of the 532, superinvolution of the, following trachelorrhaphy 538.
Utero-sacral and broad ligaments—a systematic stretching for shortening of 75.
Uterine surgery—the knife in, its use and abuse 266.

V

Vaginal hysterectomy—report of three cases 398.
Vicarious menstruation 109.
Vomiting in pregnancy—uncontrollable, cured by alimentation with a tube in the œsophagus 30.

W

What shall the surgeon do with the urethra and testes in cases of amputation and ablation of the penis? 730.

Y

Yellow fever 444, investigations relating to the etiology and prophylaxis of 246-249, observations on, with special reference to diagnosis, prognosis and treatment 411, security from 504, the panic 605.

